# Enterohemorrhagic *E. coli* (EHEC)—Secreted Serine Protease EspP Stimulates Electrogenic Ion Transport in Human Colonoid Monolayers

**DOI:** 10.3390/toxins10090351

**Published:** 2018-09-01

**Authors:** C. Ming Tse, Julie G. In, Jianyi Yin, Mark Donowitz, Michele Doucet, Jennifer Foulke-Abel, Fernando Ruiz-Perez, James P. Nataro, Nicholas C. Zachos, James B. Kaper, Olga Kovbasnjuk

**Affiliations:** 1 Department of Medicine, Division of Gastroenterology & Hepatology, Johns Hopkins University School of Medicine, Baltimore, MD 21205, USA; mtse@jhmi.edu (C.M.T.); jgin@salud.unm.edu (J.G.I.); jianyi.yin@outlook.com (J.Y.); mdonowit@jhmi.edu (M.D.); mdoucet2@jhmi.edu (M.D.), jfoulke@jhmi.edu (J.F.-A.); nzachos1@jhmi.edu (N.C.Z.); 2 Department of Internal Medicine, Division of Gastroenterology & Hepatology, University of New Mexico, Albuquerque, NM 87131, USA; 3 Department of Pediatrics, University of Virginia School of Medicine, Charlottesville, VA 22908, USA; FR3D@hscmail.mcc.virginia.edu (F.R.-P.); JPN2R@hscmail.mcc.virginia.edu (J.P.N.); 4 Department of Microbiology & Immunology, University of Maryland School of Medicine, Baltimore, MD 21201, USA; JKaper@som.umaryland.edu

**Keywords:** EHEC, serine protease EspP, human colonoid monolayers, SPATEs, diarrhea, short circuit current, CFTR, intracellular Ca^2+^

## Abstract

One of the characteristic manifestations of Shiga-toxin-producing *Escherichia coli* (*E. coli*) infection in humans, including EHEC and *Enteroaggregative E. coli* O104:H4, is watery diarrhea. However, neither Shiga toxin nor numerous components of the type-3 secretion system have been found to independently elicit fluid secretion. We used the adult stem-cell-derived human colonoid monolayers (HCM) to test whether EHEC-secreted extracellular serine protease P (EspP), a member of the serine protease family broadly expressed by diarrheagenic *E. coli* can act as an enterotoxin. We applied the Ussing chamber/voltage clamp technique to determine whether EspP stimulates electrogenic ion transport indicated by a change in short-circuit current (Isc). EspP stimulates Isc in HCM. The EspP-stimulated Isc does not require protease activity, is not cystic fibrosis transmembrane conductance regulator (CFTR)-mediated, but is partially Ca^2+^-dependent. EspP neutralization with a specific antibody reduces its potency in stimulating Isc. Serine Protease A, secreted by *Enteroaggregative E. coli*, also stimulates Isc in HCM, but this current is CFTR-dependent. In conclusion, EspP stimulates colonic CFTR-independent active ion transport and may be involved in the pathophysiology of EHEC diarrhea. Serine protease toxins from *E. coli* pathogens appear to serve as enterotoxins, potentially significantly contributing to watery diarrhea.

## 1. Introduction

There are several *E. coli* pathotypes that can cause enteric disease in humans, including enterohemorrhagic *E. coli* (EHEC), enteropathogenic *E. coli* (EPEC), enteroaggregative *E. coli* (EAEC), and enterotoxigenic *E. coli* (ETEC). Shiga toxin (Stx)-producing EHEC is one of the major agents of foodborne diarrheal disease in the US, producing ~265,000 illnesses, ~3000 hospitalizations, and ~30 deaths annually [1,2]. EHEC colonization of the human colon leads to watery diarrhea often followed by hemorrhagic colitis and, in ~10% of patients, life-threatening extra-intestinal complications that include hemolytic uremic syndrome (HUS). While Stx1 and 2 contribute to the development of hemorrhagic colitis and HUS, these virulence factors have never been shown to directly stimulate colonic water secretion [3,4].

EPEC has many similarities with EHEC, including the production of the attaching and effacing (A/E) histopathology on intestinal epithelial cells, which is characterized by intimate attachment of bacteria to the host cell plasma membrane via F-actin pedestals. The A/E lesions are mediated by the type-3 secretion system (T3SS) common to EPEC and EHEC. EPEC also causes watery diarrhea, although not hemorrhagic colitis and HUS, which is due to the lack of Stx production. Both EHEC and EPEC affect intestinal ion transporters, including Downregulated-in-adenoma (DRA), Sodium–hydrogen antiporter 3 (NHE3), and sodium–glucose linked transporter 1 (SGLT-1), all of which contribute to human diarrheal diseases. Several T3SS effector proteins have been implicated in these effects [3,5]; however, EHEC T3SS-negative strains also cause watery diarrhea [6]. Thus, the mechanism of EHEC-induced watery diarrhea has not been well defined and no enterotoxin has been identified.

A common feature among EHEC, EPEC, as well as the majority of other enteropathogenic *E. coli* and *Shigella* species is that they express the type-V secretion system involved in secretion of high-molecular-weight serine protease autotransporters of *Enterobacteriaceae* (SPATEs). A role for SPATEs in active electrogenic ion transport has been described for the EPEC Extracellular Serine Protease C (EspC), a highly potent enterotoxin that significantly increases Isc in the rat jejunum [7]. Phylogenetic analysis of the SPATEs revealed that the EHEC SPATE, EspP, is most closely related to EspC [8,9]. Sequence alignment of EspC and EspP indicates 48% amino acid identity and 65% similarity in the protease domain, as well as 45% identity and 62% similarity for the rest of the proteins including an identical catalytic site (GDSGS). This high sequence similarity between EspP and EspC predicts that EspP may also act as an enterotoxin and affect colonic epithelial ion and water transport, similar to that reported for EspC.

Recently, adult stem-cell-derived HCM have been introduced as a relevant human model to study host–pathogen interactions. HCM derived from normal human colonic crypt stem cells and grown on Transwell permeable supports allow apical exposure to enteric pathogens [10,11,12,13]. HCM in the undifferentiated state represent a deep crypt-like epithelium with a mixture of Leucine rich repeat containing G protein-coupled receptor 5 (LGR5)-enriched stem cells, transit amplifying cells and immature enterocytes and some secretory cells. Differentiated HCM contain all major cell types normally present in the colonic epithelium, including colonocytes, entero-endocrine and mucus-producing goblet cells. HCM allow controlled access to both apical and basolateral surfaces. These HCM features facilitate highly reproducible measurements of microbe-human epithelial interactions that are not achieved with 3D spherical Matrigel-embedded cultures due to variability in size/number of cells in each colonoid, limited luminal volume, and restricted luminal access. The HCM model has already provided new insights into the human pathophysiology of EHEC and EPEC [10,11,12,13] infections, whereas previous studies primarily used human colon cancer cell lines and/or animal intestinal models [14,15].

The goal of the current studies was to determine whether the EHEC serine protease, EspP, and several other SPATEs secreted by other diarrheagenic *E. coli* pathotypes, including Protease involved in colonization (Pic) and Serine protease A (SepA) of EAEC and ETEC autotransporter A (EatA), alter colonic active electrolyte transport thus potentially contributing to the diarrhea.

## 2. Results

### 2.1. EspP Demonstrates Enterotoxic Activity

**Figure 1:**
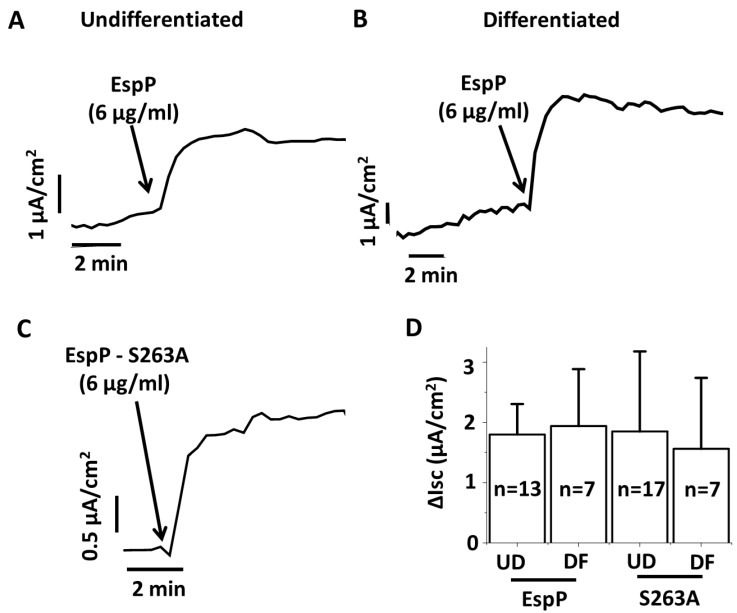
EspP stimulates Isc in proximal colonic HCM and its action is independent of its serine protease activity. (**A**,**B**) Representative Isc traces show that apically added EspP increases active ion transport in (**A**) undifferentiated and (**B**) differentiated HCM. (**C**) Representative Isc trace shows that EspP S263A also stimulates active ion transport in HCM. (**D**) Quantitative analysis of the increase in Isc shows that both EspP and EspP S263A–stimulated currents are of similar magnitude regardless of HCM differentiation status. Blue arrows indicate time that EspP or EspP S263A were added. N is the number of monolayers studied.

Enterocytes of colonic crypts are considered the main contributors to changes in ion and water transport leading to diarrhea [16,17,18]. To determine whether EspP might act as enterotoxin, the undifferentiated (UD) crypt-like HCM were treated apically with recombinant EspP [19,20] and changes in electrogenic ion transport were studied by the Ussing chamber/voltage clamp technique. EspP caused a rapid and significant increase in Isc (Figure 1A), indicating stimulation of active electrogenic intestinal ion transport. Apical EspP also induced an increase in Isc in differentiated (DF) HCM that was similar to the increase in UD HCM (Figure 1B). These results suggest that both crypt and colonic surface enterocytes likely contribute to EspP-induced changes in active colonic electrolyte transport in disease.

### 2.2. EspP Enterotoxic Activity is Independent of its Serine Protease Activity

EspP is a very potent cytotoxin and damages the intestinal epithelial cell brush border as well as causes cell death [20,21,22]. The cytotoxicity of EspP is due to its serine protease activity. We used the EspP serine protease-inactive mutant, EspP S263A, described previously in detail [10,23], to test the role of EspP enzymatic activity on Isc stimulation. Surprisingly, apical treatment of HCM with EspP S263A caused a rapid and significant increase in Isc in both UD (Figure 1C) and DF cultures (Figure 1D). The ΔIsc was similar in both EspP and EspP S263A treatments and independent of HCM differentiation. These data demonstrate that EspP enterotoxic activity might affect both colonic crypt and surface epithelia and is independent of its cytotoxic and protease activity.

### 2.3. EspP Specifically Alters Isc in a Concentration-Dependent Manner

**Figure 2:**
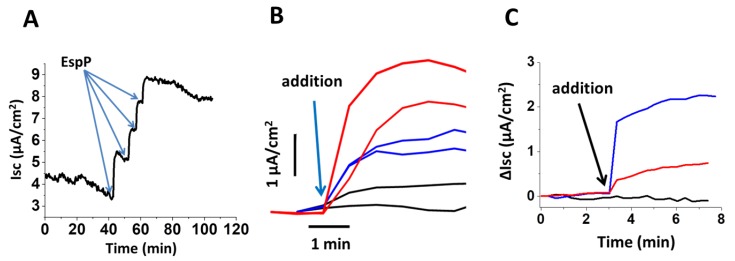
Isc stimulated by EspP in proximal HCM is concentration dependent and EspP-specific. (**A**) EspP-stimulated Isc increases with each sequential 6 µg/mL apical addition of EspP (blue arrows). (**B**) Representative Isc traces show that apically added purified recombinant EspP (6 µg/mL; red traces) as well as lysate from AD202 bacteria containing EspP plasmid (30 µL, blue traces), but not lysate from AD202 parental strain lacking either EspP or EspP S263A plasmid (30 µL, black traces), rapidly increase Isc. (**C**) Representative Isc traces show that pre-absorbing EspP toxin with specific anti-EspP antibodies reduced the EspP-increase in Isc by ~70% (red traces); black trace—time control; blue trace—purified EspP. Blue arrows indicate the time point when toxins or lysates were added apically.

EspP-stimulated Isc increased with increasing concentrations of apically added EspP (Figure 2A). To further test whether the EspP- or EspP S263A-stimulated increase in Isc in HCM is specific and not induced by other factors secreted by the AD202 *E. coli* strain, from which the recombinant EspP or EspP S236A were harvested [19], HCM were apically exposed to either detergent-free lysate of EspP-expressing AD202 bacteria or to lysate of the parental AD202 strain lacking either EspP or EspP S263A plasmid. The lysate from the EspP-expressing AD202 bacteria, but not from the parental strain, stimulated the Isc with a magnitude less than that stimulated by recombinant purified EspP (Figure 2B). Also, EspP toxin pre-incubated with a specific anti-EspP antibody [10,19] substantially decreased the Isc in HCM (Figure 2C), further confirming that EspP, is the main contributor to the increased ion transport.

### 2.4. EspP-Stimulated Current is CFTR-Independent

**Figure 3:**
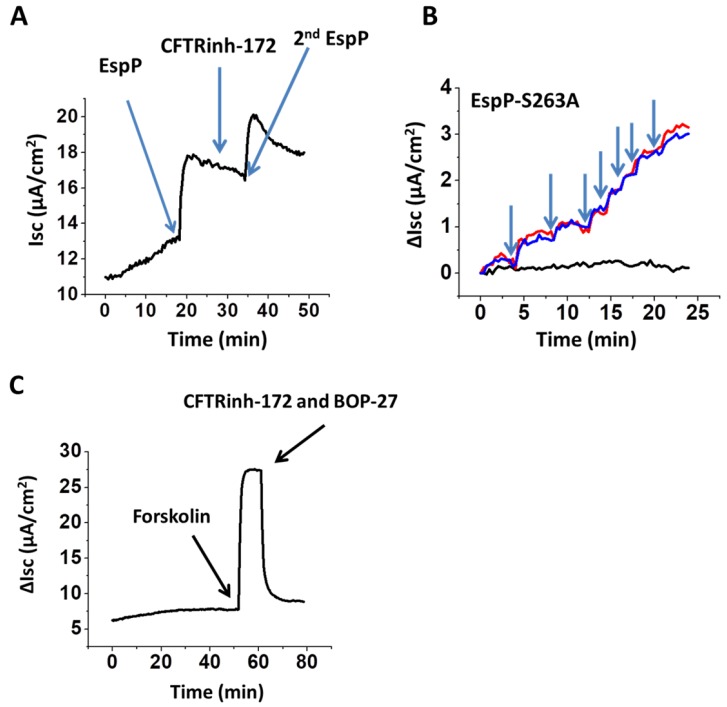
EspP-stimulated Isc in proximal HCM is CFTR-independent. Representative (**A**) trace of EspP-stimulated Isc shows that apical addition of CFTR inhibitor CFTR_inh_-172 (25 µM) neither inhibits Isc nor prevents further Isc increase following second EspP addition (each EspP addition is 6 µg/mL). (**B**) Traces of Isc increase following the escalating apical addition of EspP S263A (each addition is 6 µg/mL) in the presence (red) and the absence of (blue) combination of CFTR inhibitors, CFTR_inh_-172 (25 µM) and BPO-27 (5 µM). Time control (black) in the absence of toxin. (**C**) Forskolin (10 µM) rapidly generates Isc, which is completely inhibited by a combination of CFTR inhibitors, used in (**B**). Blue arrows in all panels indicate the time-point of corresponding apical treatment.

The molecular mechanisms of many acute secretory diarrheas caused by bacterial enterotoxins, including cholera toxin, is due to activation of CFTR, the main epithelial Cl^−^ channel expressed at the apical membrane of enterocytes or colonocytes. Next, we tested whether the EspP-stimulated Isc is CFTR-mediated. We used two different CFTR inhibitors, a widely used specific inhibitor CFTR_Inh_-172 alone or in combination with CFTR inhibitor BPO-27 [24,25,26], that was recently reported to be more potent and specific than CFTR_Inh_-172. Neither inhibitor affected the EspP-(Figure 3A) or EspP S263A-stimulated Isc (Figure 3B) in either DF or UD HCM, indicating that the EspP-stimulated Isc is CFTR independent.

Like EspP, the effect of the EspP S263A mutant on Isc was also concentration dependent. Moreover, the presence of CFTR inhibitors did not prevent toxin concentration-dependent increase in Isc. To confirm the presence of functional CFTR in our proximal colonoid cultures, we used forskolin, a classical CFTR activator that elevates intracellular cAMP concentrations via adenylyl cyclase activation [27]. Forskolin generated a rapid increase in Isc, which was completely inhibited by a combination of CFTR inhibitors, CFTR_inh_-172 and BPO-27 (Figure 3C), as expected, indicating that HCM express functional CFTR. Taken together, these data demonstrate that CFTR is not responsible for EspP-induced changes in active electrogenic ion transport in proximal HCM.

### 2.5. EspP-Induced Isc is Partially Ca^2+^ Dependent

**Figure 4:**
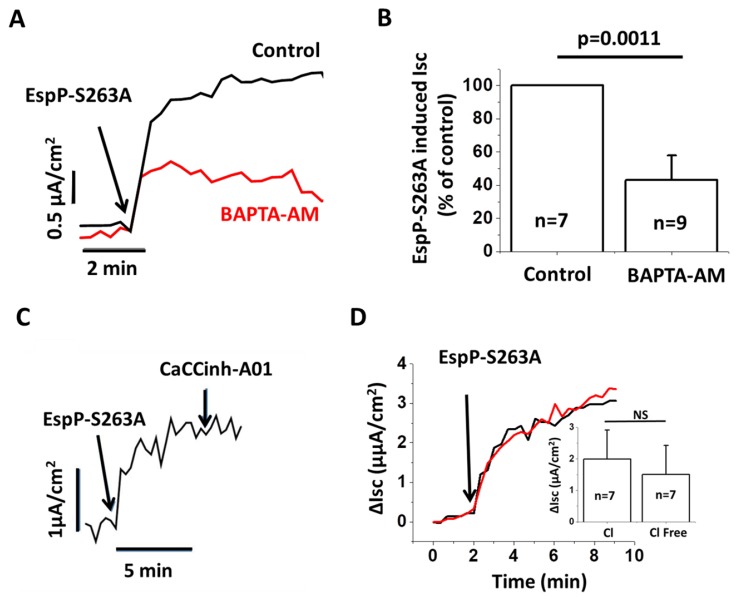
EspP S263A-stimulated Isc in proximal HCM is Ca^2+^-dependent. (**A**) Representative traces and (**B**) quantitative analysis of BAPTA-AM effect on EspP S263A-stimulated Isc. BAPTAM-AM (25 µM pretreatment for up to 30 min) significantly inhibits EspP S263A-stimulated Isc. (**C**) EspP S263A-stimulated Isc is not inhibited by CaCC_inh_-A01. (**D**) Esp S263A-stimulated Isc is similar in the buffer containing Cl^−^ (black trace) and without Cl^−^ (red trace). The inset shows that ΔIsc is quantitatively similar in Cl^−^ and Cl^−^-free buffer; n is the number of monolayers studied.

We next tested the hypothesis that EspP-induced changes in active ion transport could be Ca^2+^ dependent. Indeed, pretreatment of HCM with the cell-permeable calcium chelator BAPTA-AM significantly (~60%) inhibited the Isc generated by EspP S263A (Figure 4A,B). These data indicate that Ca^2+^-activated chloride channels (CaCC) may mediate EspP response [28]. However, the CaCC inhibitor, CaCC_inh_-A01 failed to inhibit EspP stimulated Isc (Figure 4C). Furthermore, treatment of HCM with EspP in Cl^−^-free solution did not prevent the EspP effect on Isc (Figure 4D), indicating that EspP-stimulated current is not Cl^−^-dependent. Thus, CaCCs are not mediating EspP-stimulated secretion even though EspP stimulates a Ca^2+^-dependent change in active ion transport in HCM.

### 2.6. Effects of Class-2 SPATEs on Ion Secretion by Human Intestinal Epithelial Cultures

The general structure of SPATEs is comprised of three functionally different domains: the signal peptide, which targets the protein to the bacterial periplasm; the pore-forming C-terminal translocator domain, which targets the protein to the bacterial outer membrane; and the N-terminal passenger domain, which encodes the characteristic serine protease motif (GDSGS) on SPATE proteins along with the residues involved in the formation of the catalytic triad (His, Asp and Ser) [29,30,31]. Phylogenetic analysis of the amino acid sequence of the SPATE passenger domain reveals two distinct classes of SPATE proteins: class-1, cytotoxic; and class-2, lectin-like immunomodulators.

**Figure 5:**
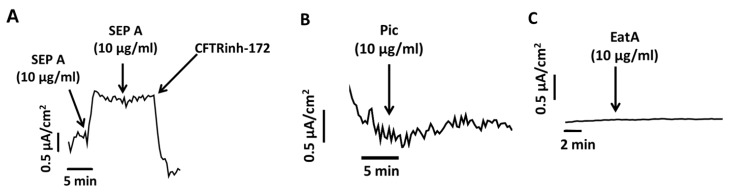
Effect of other SPATEs on active ion transport in HCM and human enteroid monolayers (HEM). (**A**) SepA—stimulated ion secretion is CFTR-mediated and completely inhibited by CFTR_inh_-172 (25 µM). Blue arrows show the time points of each SepA addition (10 µg/mL) as well as addition of CFTR_inh_-172. Neither Pic (10 µg/mL) (**B**) nor EatA (10 µg/mL) (**C**) stimulated Isc in proximal UD HCM or jejunal HEM, respectively. Blue arrows indicate the time points of Pic or EatA addition.

Both EHEC-secreted EspP and EPEC-secreted EspC that stimulate intestinal epithelial ion secretion belong to class-1 cytotoxins, while the EAEC-secreted Pic and SepA and ETEC-secreted EatA are of the immunomodulatory class-2 SPATEs. We next tested whether Pic, SepA and EatA also exert effects on ion transport in human colonoid cultures. Similar to EspP, apical treatment of HCM with recombinant SepA (10 µg/mL) rapidly and significantly increased Isc (Figure 5A). Unlike EspP, the ΔIsc reached a maximum response at 10 µg/mL SepA. Importantly, the SepA-stimulated current was a Cl^−^ secretory process via CFTR activation and was completely inhibited by CFTR_inh_-172.

In contrast to SepA, recombinant Pic failed to induce Isc in UD HCM (Figure 5B). Similarly, treatment of human enteroid monolayers (HEM) derived from the jejunum, the major intestinal site of ETEC infection, with EatA did not alter Isc (Figure 5C). We conclude that both Pic and EatA failed to directly stimulate Isc when added to the apical surfaces of HCM and HEM, respectively. In contrast, SepA significantly contributed to Cl^−^ secretion in a CFTR-dependent manner, and thus may contribute to EAEC-induced diarrhea.

## 3. Discussion

Despite significant efforts to eradicate infectious diarrheal diseases, large-scale epidemics and the emergence of novel mutant strains continue to affect populations worldwide. Recent major incidents include the 2010 cholera outbreak due to *Vibrio cholerae* in Haiti and the 2016 outbreak in Yemen [32,33], and foodborne illnesses across Europe in 2011 caused by a novel EAEC O104:H4 strain that had acquired Stx2a [34,35]. Although antibiotic treatment is usually the first and most effective therapy for bacterial GI infections, such measures against STEC infection can exacerbate disease, yielding extra-intestinal complications such as hemolytic uremic syndrome [14,36,37,38,39,40]. In addition, anti-motility agents such as loperamide are also contra-indicated for STEC infection. Thus, the only treatment option currently available is non-specific rehydration therapy and other supportive therapies.

A limitation in developing new therapeutic options to treat diarrhea due to EHEC is a full understanding of the bacterial factors that contribute to diarrhea. Here we used the human adult stem cell- derived ex vivo colonic epithelium cultures termed colonoids as a novel model to test the role of SPATEs, a class of bacterial effectors that share the property of having serine protease activity, in the development of watery diarrhea. Compared to well characterized colonic carcinoma- derived intestinal epithelial cell lines, colonoids provide many advantages [11,41]. They are established from biopsies obtained from healthy donors and the resulting cultures preserve the donor’s genotype/phenotype as well as intestinal segmental specificity. Colonoid cultures include a mixture of all types of intestinal epithelial cells at close to physiologic ratios, including LGR5^+^ stem cells, colonocytes, goblet, and enteroendocrine cells [42,43]. Transcriptome and immunofluorescence analyses show that undifferentiated cultures grown in the presence of high concentrations of wingless-related integration site (Wnt) 3A, R-spondin-1 and Noggin represent crypt-like epithelium, while differentiated cultures maintained in the absence of Wnt3A and R-spondin-1 develop into surface-like epithelium with a thick layer of attached mucus [10,11,42,43]. Thus, colonoids are a physiologically relevant human intestinal epithelial model to determine the contribution of colonic crypt-like epithelium vs. surface-like epithelium to the net ion secretion in physiologic and pathophysiologic conditions, as in diarrhea induced by pathogenic microbes. Importantly, when maintained in an appropriate mixture of growth factors, these UD cultures propagate indefinitely allowing multiple experimental repetitions [11,42,43,44].

Using this HCM model, we show that EHEC-secreted EspP acts as an enterotoxin and stimulates Isc in a toxin concentration-dependent manner. Pre-absorption with a specific antibody significantly reduced EspP-dependent stimulation of Isc, further indicating the specificity of EspP-induced transepithelial current. Surprisingly, EspP-induced Isc was not mediated by CFTR or CaCCs, but was partially Ca^2+^-dependent. Further studies are needed to determine the specific active transport process affected by EspP and cellular transporter(s) involved in EspP-stimulated transepithelial current.

The EspP protein motif responsible for enterotoxigenic activity remains to be identified, because EspP-stimulated Isc did not require its protease activity. These data indicate that SPATEs are multifunctional virulence factors and that protease activity is not always the dominant function in pathogenetic mechanisms attributed to these proteins [29,30,31]. Indeed, EAEC O104:H4, in addition to Stx, acquired a rare combination of SPATEs genes, *sepA*, *sigA*, and *pic* [45,46]. Although Pic did not exhibit enterotoxigenic activity in our studies, confirming published observations [47], SepA stimulated Cl^−^ secretion in a CFTR-dependent manner in HCM. Thus, SepA may contribute to EAEC O104:H4-induced diarrhea in patients. Additionally, EAEC SPATEs were shown to be important for robust colonization and subsequent induction of diarrheal disease in an infant rabbit model of EAEC O104:H4 infection [48] further indicating the multi-functionality of this class of bacterial proteins. Since diarrhea producing bacteria often make multiple SPATEs, studies of their contributions both alone and in combination will be required to better understand the pathophysiology of diarrhea; and it is expected that systems such as the human enteroid monolayers, as used here, will allow such experimental approaches to be successfully undertaken.

The clinical manifestation of watery diarrhea in EHEC patients is substantially milder (both stool volume and number of bowel movements) compared to *Vibrio cholera-*induced massive water diarrhea, which can reach up to 1 L per hour. *V. cholera*-induced rate of fluid loss is not typically observed in other cases of diarrheal illness [49]. Thus, our data that the Isc amplitude generated by EspP is substantially lower compared to the Isc generated by forskolin, which functions similarly to cholera toxin activation of CFTR, correctly reflects the clinical differences between EHEC and *V. cholera-*induced watery diarrhea.

The significant difference in water loss between *V. cholera* and EHEC-induced watery diarrhea is also reflected in disease management. Rehydration is the first line of treatment in the case of *V. cholera-*induced diarrhea, but currently not in the case of EHEC-infected patients. However, this small dehydration may have severe consequences in EHEC-induced disease progression. Recent clinical data analysis shows that the hydration status of EHEC-infected patients has a significant effect on disease outcome. Dehydration is associated with worse outcomes, including the development of oligo-anuric renal failure in children with HUS, need for renal replacement therapy, central nervous system involvement and death [50]. Thus, understanding the molecular mechanisms of EHEC-induced watery diarrhea, including the role of EspP in this process, may provide a better outcome for EHEC-caused systemic diseases.

In addition, it is known that increases in second messengers in intestinal cells have synergistic effects on Cl-secretion. We hypothesize that the small increase in Isc caused by EspP is probably only a component that drives fluid secretion in EHEC diarrhea, although the additional complexity of the effects of multiple secretagogues, including EspP, has not been evaluated.

## 4. Conclusions

EHEC-secreted virulence factor EspP acts as an enterotoxin and causes change in active ion transport in HCM in a toxin concentration-dependent manner. EspP-induced current is not mediated by CFTR, is not Cl^−^-secretory, but it is partially Ca^2+^-dependent and may contribute to the pathophysiology of EHEC diarrhea. This is the first enterotoxin identified in EHEC and a newly recognized function of EspP, which is independent of its protease activity.

## 5. Materials and Methods

### 5.1. Reagents

All reagents, unless otherwise specified, including CFTR inhibitor CFTRinh172, CaCC inhibitor and BAPTA-AM were purchased from Sigma-Aldrich (St. Louis, MO, USA). CFTR inhibitor BPO-27 was a generous gift from Dr. Alan Verkman, USCF. Anti-EspP antibody was a generous gift from Dr. Harris D. Bernstein, NIDDK.

### 5.2. Purification of EspP and EspP S263A

Genes encoding full-length EspP and serine protease mutant EspP S263A were cloned into the pRLS5 plasmid under the control of an IPTG-inducible promoter as previously described [51]. The plasmids were transformed into the *E. coli* strain AD202. Colonies from the EspP- or EspP S263A—containing AD202 bacteria were inoculated into Luria Broth (LB) with 100 μg/mL ampicillin for overnight cultures. The cultures were spun down and cells washed twice in fresh LB. The resuspended bacteria were transferred to LB media and EspP or EspP S263A was expressed upon IPTG induction. The proteins were precipitated from the supernatant with 60% ammonium sulfate. The recombinant proteins were concentrated by centrifugation (Amicon Ultra 15 mL, MWCO 30 kDa; Millipore Sigma, St. Louis, MO, USA), solubilized in PBS and filtered using 0.22 µm syringe filters. Final protein concentration was determined by Bradford protein assay (Bio-Rad, Hercules, CA, USA). Serine protease activity of recombinant EspP and lack of activity of EspP S263A was verified by pepsin cleavage assay [52]. Small aliquots were stored at −80 °C.

For EspP neutralization by antibody, 100 μL of purified EspP toxin was incubated with 20 µL of specific anti-EspP antibodies overnight at 4 °C. Antibody-EspP complexes and excess EspP antibodies were removed with Protein A/G Plus Agarose (Santa Cruz sc-2003, Paso Robles, CA, USA) by incubating the solution for 2 h and centrifugation at 1000 g for 5 min. The EspP depleted supernatant was used for Isc studies. The levels of EspP depletion by antibody were visualized by Coomassie Blue staining of the pre-depleted and post-depleted solutions subjected to SDS/PAGE (data not shown).

### 5.3. Purification of Pic and SepA

Expression and purification of Pic and SepA proteins from minimal clones in *E. coli* HB101 have been described previously [28]. Briefly, overnight Luria broth cultures of *E. coli* HB101 expressing SepA or Pic clones were centrifuged at 7500× *g* for 10 min. Culture supernatants were passed through a 0.22 μm-pore size filter to remove residual bacteria and concentrated with 30-kDa-cutoff spin filter devices (Millipore, Bedford, MA, USA). The supernatants were then separated by anion exchange chromatography using Sepharose Q resin and stepwise elution. Fractions containing SPATEs were again concentrated as described above with spin filters. Anion-exchange purified proteins were treated with Endo-Trap-Blue (Hyglos GmbH, Bernried am Starnberger See, Germany) to remove LPS accordingly to the manufacturer’s specifications, dialyzed in 1 × PBS pH 7.0, and stored in small aliquots at −80 °C.

### 5.4. Human Colonoid and Enteroid Cultures

Human colonoid and enteroid cultures were established from de-identified tissue samples obtained after endoscopic or surgical procedures from four different subjects that provided informed consent utilizing the previously described methods [10,44]. All experimental protocols were approved by the JHU Institutional Review Board (IRB# NA_00038329, approval date: 27 February 2014). All methods were carried out in accordance with approved guidelines and regulations. Briefly, human cultures were generated from isolated intestinal crypts embedded in Matrigel (Corning, Tewksbury, MA, USA) in 24-well plates and cultured in the presence of Wnt3A, R-spondi-1 and Noggin containing undifferentiated media (UDM), as previously described in detail [10,12]. Matrigel cultures in UDM media were used for the propagation of colonoids and enteroids. To generate the monolayer cultures, colonoids or enteroids were triturated in Cultrex Organoid Harvesting Solution (Trevigen, Gaithersburg, MD, USA), and the fragments were collected by centrifugation and resuspended in UDM. Enteroid fragments (100 μL) were seeded onto 0.4 μm pore polyester (PET) membrane 24-well cell culture inserts (Transwell; Corning, Tewksbury, MA, USA) pre-coated with human collagen IV (34 μg/mL; Millipore Sigma, St. Louis, MO, USA). Monolayers were cultured in UDM at 37 °C, 5% CO_2_. Under these conditions, cultures reached confluency in 7–14 days. Confluent monolayers were differentiated for 5 days in media free of Wnt3A and R-spondin-1.

### 5.5. Short-Circuit Current Measurements with the Ussing Chamber/Voltage Clamp Technique

Short-circuit current (Isc) in HCM was measured as previously described [25,26]. Unless indicated otherwise, the concentration of EspP and EspP S263A added to apical surfaces was 6 µg/mL. Transwell inserts seeded with human colonoid and enteroid monolayers were mounted in Ussing chambers (Physiological Instruments, San Diego, CA, USA). The apical and basolateral hemichambers were filled with Krebs-Ringer bicarbonate (KBR) buffer that was gassed continuously with 95% O_2_/5% CO_2_, maintained at 37 °C, and connected to a voltage-current clamp apparatus (Physiological Instruments) via Ag/AgCl electrodes and 3 M KCl agar bridges. The KRB buffer was made of 115 mM NaCl, 25 mM NaHCO_3_, 0.4 mM KH_2_PO_4_, 2.4 mM K_2_HPO_4_, 1.2 mM CaCl_2_, 1.2 mM MgCl_2_, pH 7.4. To make Cl^−^-free buffer, Cl^−^ in KRB buffer was replaced with equimolar gluconate. Cl^−^-free buffer was supplemented with additional 5 mM calcium gluconate to maintain the level of free calcium. Additionally, buffer in the basolateral hemichamber was supplemented with 10 mM glucose as an energy substrate; buffer in the apical hemichamber was supplemented with 10 mM mannitol to maintain the osmotic balance. Current clamping was employed and short-circuit current was recorded every 20 s by the Acquire & Analyze software 2.2.2 (Physiological Instruments, San Diego, CA, USA). To investigate cAMP-stimulated anion secretion, 10 μM of forskolin was added to the basolateral hemichamber, followed by addition of the CFTR inhibitors.
